# Simple, sensitive and rapid determination of linifanib (ABT-869), a novel tyrosine kinase inhibitor in rat plasma by UHPLC-MS/MS

**DOI:** 10.1186/1752-153X-8-13

**Published:** 2014-02-17

**Authors:** Muzaffar Iqbal, Essam Ezzeldin, Tanveer A Wani, Nasr Y Khalil

**Affiliations:** 1 Department of Pharmaceutical Chemistry, College of Pharmacy, King Saud University, P.O. Box 2457, Riyadh 11451, Saudi Arabia; 2 Bioavailability Laboratory, College of Pharmacy, King Saud University, P.O. Box 2457, Riyadh, Saudi Arabia

**Keywords:** Linifanib, ABT**-**869, UHPLC**-**MS**/**MS, Rat plasma, Pharmacokinetics

## Abstract

**Background:**

Linifanib (ABT-869) is an orally active receptor tyrosine kinase inhibitor, which simultaneously inhibits vascular endothelial and platelet derived growth factor receptor. The aim of the present study was to develop an UHPLC-MS/MS method for the quantification of linifanib in rat plasma to support the pharmacokinetic and toxicokinetic studies.

**Results:**

Linifanib was separated on Acquity UPLC BEH™ C_18_ column (50 × 2.1 mm, i.d. 1.7 μm) using acetonitrile-10 mM ammonium acetate (60:40, v/v) as an isocratic mobile phase at a flow rate of 0.3 mL/min with sunitinib as internal standard (IS). Detection was performed on tandem mass spectrometer using electrospray ionization source in positive mode by multiple reaction monitoring. The monitored transitions were set at m/z 376.05 > 250.97 for linifanib and m/z 399.12 >283.02 for IS, respectively. Both linifanib and IS were eluted at 0.68 and 0.44 min, respectively with a total run time of 2.0 min only. The calibration curve was found to be linear over the concentration range of 0.40–500 ng/mL. The intra- and inter-day precision value was ≤10.6% and the accuracy ranged from 90.9-108.9%. In addition, all the validation results were within general assay acceptability criteria according to guidelines of bio-analytical method validation.

**Conclusion:**

A selective and sensitive UHPLC-MS/MS method was developed and validated for the determination of linifanib in rat plasma for the first time. The developed method is simple, sensitive and rapid in terms of chromatographic separation and sample preparation and was successfully applied in a pilot pharmacokinetic study in rats.

## Background

Vascular endothelial and platelet derived growth factor receptor (VEGFR and PDGFR) plays a major role in angiogenesis and tumor cell proliferation. It has been reported that the simultaneous inhibition of these two receptors achieves greater antitumor activities than inhibition of either receptor alone [1,2]. Linifanib (ABT-869) is an orally active novel small molecule multi-target receptor tyrosine kinase (RTK) inhibitor, which simultaneously inhibits VEGFR and PDGFR with minimal activity against unrelated RTKs. It has potent inhibitory activity against VEGFR-1, VEGFR-2, PDGFRb, colony-stimulating factor 1 receptor, and fms-related tyrosine kinase 3, with minimal activity against unrelated tyrosine and serine/threonine kinases [3-5]. Linifanib has shown prominent antitumor activity against solid tumors in phase 2 studies e.g. non-small cell lung cancer, hepatocellular carcinoma and renal cell carcinoma [6-9] and presently are in phase 3 studies in patients with hepatocellular carcinoma [9].

Based on the available preliminary phase I study data, the pharmacokinetics of linifanib was dose proportional and time invariant. It was rapidly absorbed with peak plasma concentration achieved in approx. 2 h across all dose levels. The oral clearance was 2.7 L/h, and the main systemic metabolite was the carboxylate metabolite and only 15% of the dose was recovered as unchanged in urine [10,11].

Considering linifanib as a new potential antitumor drug, a selective and sensitive bioanalytical method is required for its pharmacokinetic and toxicokinetic studies. The chromatographic separation procedure of reported LC-MS/MS method (for linifanib and its acid metabolite) in plasma requires pre-column back wash and takes more than 6 min to complete the one sample analysis [12]. Though its extraction procedure was based on automated liquid liquid extraction (high-throughput), but mean extraction recovery was only 18% and it also required Hamilton automated liquid handler with 96 well plates for operation. Wu H et al 2008, used salting-out assisted liquid/liquid extraction procedure, but the recovery couldn’t exceeded of 40% [13]. Amongst the available analytical techniques, UHPLC has gained a considerable attention due to use of Acquity BEH column, which not only increased the separation throughput and efficiency but also reduced the retention time and volume of solvent required during separation [14-16]. The aim of the present study was to develop a sensitive UHPLC-MS/MS method, which can facilitate the rapid determination of linifanib in plasma using simple chromatographic separation and sample preparation procedure.

## Experimental

### Chemicals and reagents

**Figure 1:**
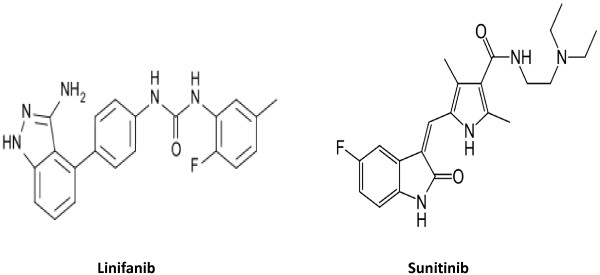
Chemical structure of linifanib and sunitinib.

Linifanib (purity ≥98%) was purchased from Weihua Pharma Co., Limited, Zhejiang, China and sunitinib malate (internal standard; purity ≥98%) was purchased from Sigma-Aldrich, USA (Figure 1). Methanol and acetonitrile were of HPLC grade obtained from Winlab Laboratory, whereas formic acid and ammonium acetate were of analytical grade obtained from BDH Laboratory, England. All aqueous solutions used in this study were obtained from Milli-QR Gradient A10R (Millipore, Moscheim Cedex, France) having pore size 0.22 μm. Blank rat plasma was separated from the healthy rats which obtained from the Animal Care and Use Centre, College of Pharmacy, King Saud University, Riyadh, Saudi Arabia.

### Preparation of stock solutions, calibration standards and quality control samples

Standard stock solutions of 1.0 mg/mL were prepared for linifanib in methanol and sunitinib (IS) in dimethyl sulphoxide by dissolving the accurate amounts of their reference standards. A series of working standard solutions of linifanib were prepared by further dilution of the stock solution using acetonitrile. All stock solutions and working solutions were kept in refrigerator at 4°C. Plasma calibration standards were prepared by spiking the appropriate amounts of the working standard solutions into blank plasma to obtain final concentration levels of 0.40, 1.13, 3.24, 10.80, 36.0, 120, 300 and 500 ng/mL. Quality control (QC) samples at four different concentrations: 0.45, 1.28, 42.50 and 425 ng/mL in blank rat plasma were also prepared in a similar manner and treated as LOQ QC, LQC, MQC and HQC respectively. Both plasma calibration standards and QC samples were kept at −80°C until used during validation and/or sample analysis. The IS working solution (1 μg/mL) for routine use was prepared by diluting the IS stock solution in acetonitrile and were stored at 4°C.

### UHPLC separation and MS/MS parameters

The equipment consisted of ACQUITY™ UHPLC system coupled to triple-quadruple tandem mass spectrometer (Waters Corp., Milford, MA, USA). The UHPLC system consisted of quaternary solvent manager, a binary pump, degasser, autosampler with an injection loop of 10 μL and a column heater-cooler. The chromatographic separation was performed on Acquity BEH™ C_18_ column (50 × 2.1 mm, i.d., 1.7 μm, Waters, USA) maintained at ambient temperature. The mobile phase was comprised of acetonitrile:10 mM ammonium acetate (60:40, v/v) at a flow rate of 0.3 mL/min. Linifanib and IS were eluted at 0.68 and 0.44 min respectively with a total run time of 2.0 min only. The injection volume was 5 μL in partial loop mode and the temperature of the autosampler was 10°C.

Triple-quadruple tandem mass spectrometer equipped with electrospray ionization (ESI) interface was used for analytical detection. The detection was obtained in ESI positive mode using multiple reaction monitoring (MRM), with transition channels of proton molecular ions was m/z 376.05 > 250.97 for linifanib and m/z 399.12 >283.02 for IS, having dwell time of 0.106 s. Nitrogen was used as a desolvating gas at a flow rate of 600 L/h. The desolvating temperature was 350°C whereas source temperature was 150°C. The collision gas (argon) flow was 0.1 mL/min and capillary voltage was set at 3.5 kV. The compound parameters like cone voltage and collision energy were set at 46 V & 30 eV for linifanib and 36 V & 22 eV for IS respectively. The Mass Lynx software (Version 4.1, SCN 714) was used to control the UHPLC-MS/MS system and data was collected and processed using Target Lynx™ program.

### Sample preparation

Plasma samples stored at around −80°C were thawed, left for 1 h at room temperature and vortexed for 30 sec before extraction. In a fresh 1.5 mL centrifuge tube, 200 μL plasma (calibration standards, QCs and unknown samples) followed by 20 μL of IS (1 μg/mL) was added. The sample was vortexed for 30 sec and again by addition of 15 μL of formic acid to each tube. After vortexed, 445 μL of acetonitrile was added and again vortexed gently for 2 min, then centrifuged for 8 min at 12500 rpm at 4°C. After centrifugation, supernatant was transferred to a clean tube, and evaporated to dryness under a stream of nitrogen at approximately 40°C. The residue was reconstituted in 200 μL of the mobile phase, vortexed for 30 sec and 5 μL was injected into UPLC–MS/MS for analysis.

#### Method validation

A full method validation was performed according to guidelines set by the United States Food and Drug Administration and European Medicines Agency [17,18]. The developed method was validated in terms of selectivity, linearity of response, accuracy, precision, recovery, matrix effects, dilution integrity and stability of the analytes during both short-term sample processing and long-term storage.

Method selectivity was investigated by the comparison of chromatograms of blank plasma obtained from different healthy rats with the corresponding chromatograms of plasma samples spiked at LLOQ (0.40 ng/mL) level of the analyte and 100 ng/ml of the IS.

The linearity of the method was determined by performing the three calibration curves with eight different concentrations ranging from 0.40-500 ng/mL. The correlation coefficient r^2^ > 0.995 was desirable for all the calibration curves. The peak area ratios of the analyte to IS were calculated and the calibration curves were established by fitting these ratios to the corresponding concentrations using weighted least square linear regression. The lowest concentration of the analyte (0.40 ng/mL) on the calibration curve was considered as LLOQ with acceptable accuracy and precision.

The precision of the assay is expressed as percentage coefficient of variation (% CV), whereas accuracy is expressed as a percentage deviation from the respective nominal value. For precision and accuracy, QC samples at four different concentration levels (LOQ QC, LQC, MQC and HQC) were analyzed in six replicates on the same day (intra-day) and on three different days (inter-day) respectively. The deviation in mean value of precision should not exceed 20% for the lowest QC samples and 15% for the other QC samples and accuracy should be within ±20% for the lowest QC samples and ±15% for the other QC samples.

The extraction recovery of linifanib at three QC levels and IS at 100 ng/mL were evaluated by comparing peak area ratios of plasma spiked with analyte prior to extraction with the peak area ratios of plasma spiked with analyte after the extraction. Calculation of the matrix effects was conducted by calculating the peak area ratio of extracted rat plasma post spiked with analyte to the peak area of the analyte in reconstitution solution in the same concentration. Deviation in linifanib and IS concentrations of a maximum of 15%were considered acceptable as recommended in EMEA bioanalytical guideline [18].

The stability of linifanib in rat plasma during the sample storage and processing conditions was assessed by analyzing six replicates of LQC and HQC. Short-term stability was assessed after keeping the plasma samples at ambient temperature for ∼ 6 h, the freeze–thaw stability was determined after three cycles of freeze-thaw. Post-preparative stability was determined by storing the reconstituted QC samples for ∼ 48 h under autosampler conditions (maintained at 10°C) before being analyzed. Long-term stability was assessed after storage of the test samples at around −80°C for 30 days. The working solutions and stock solutions of linifanib and the IS were also evaluated for stability at room temperature for 12 h and at refrigerator temperature (below 10°C) for 15 days. The samples were considered stable in plasma at each concentration if the deviation from the mean calculated concentration of quality control samples were within ±15%.

Dilution integrity was evaluated to ensure the integrity of analyte in those samples which were beyond upper limit of the standard curve and need to be diluted. A fresh stock of linifanib was prepared and spiked in plasma to get a concentration level of 1.8 times of highest standard of the usual calibration standard, and then diluted 2 and 4 times with the same plasma. Six aliquots of both dilutions were processed along with freshly spiked calibration standards and analyzed by back calculation using regression equation obtained. The integrity of the samples were considered to be maintained if percentage nominal is within ± 15% of nominal values and % CVs ≤ 15% at both diluted levels.

### Application to pharmacokinetic interaction study in rats

To demonstrate the utility of this method, a pilot pharmacokinetic study of linifanib was performed in six male wistar albino rats weighting from 220 to 230 g. After an overnight fasting, all rats received linifanib (5 mg/kg, oral, dissolved in HPMC). Blood samples (approximately 0.5 mL) were collected from the retro-orbital plexus into heparinized microfuge tubes at different time interval after linifanib administration and plasma samples were harvested by centrifuging the blood at 4500 rpm for 8 min and stored frozen at −80 ± 10°C until analysis. The pharmacokinetic parameters: C_max_, T_max_, AUC, t_½_ and K_el_ were calculated using WinNonlin software.

## Result and discussion

### Method development

#### Optimization of mass spectroscopy condition

**Figure 2:**
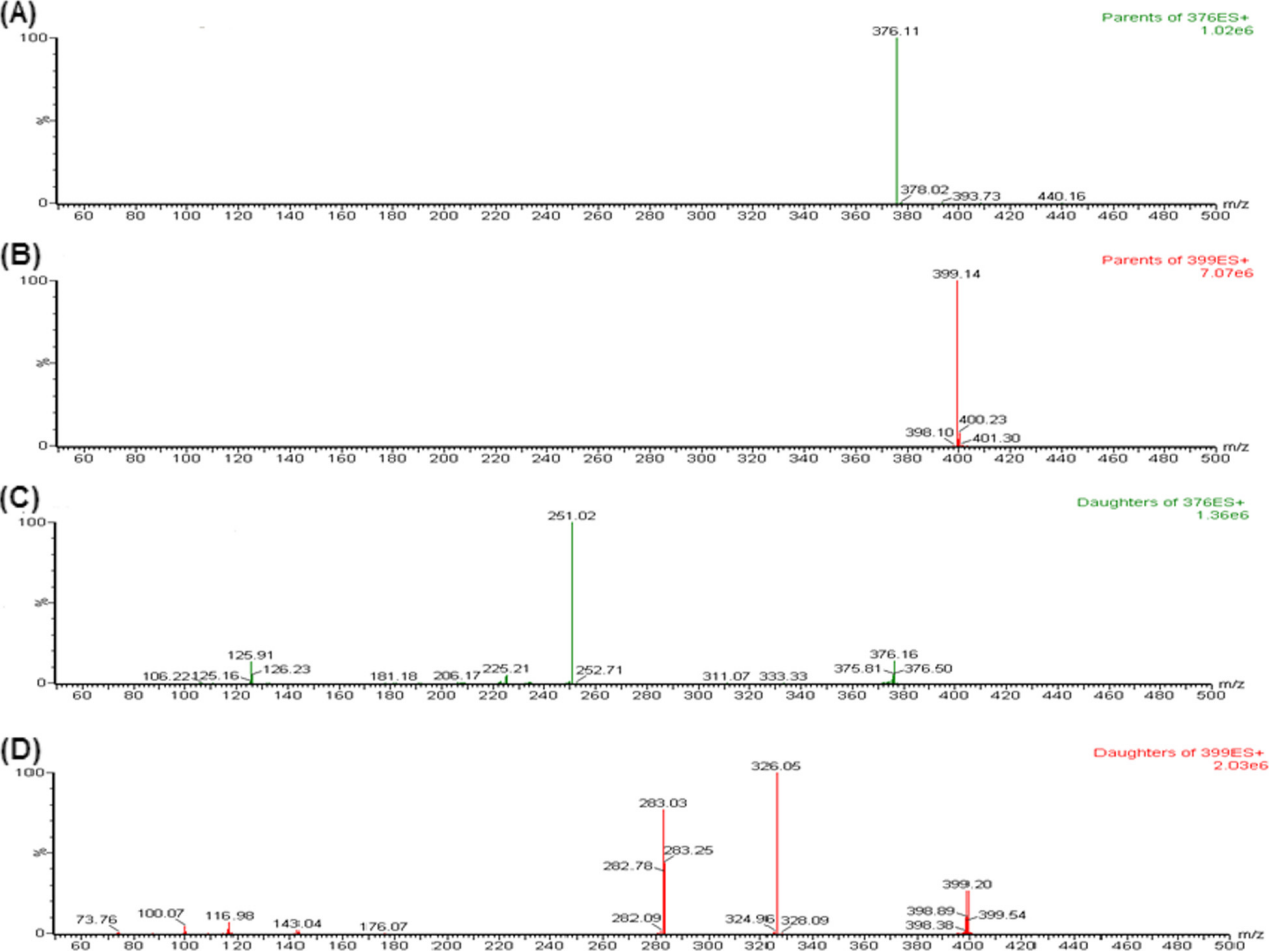
MS spectra of linifanib (A) and IS (B) and MS/MS spectra of linifanib (C) and IS (D).

Expecting high selectivity and sensitivity of the MS/MS detection, operation parameters were carefully optimized before detection by MRM mode. Initially, linifanib and IS response were evaluated by directly infusing into the mass spectrometer for tuning in both positive and negative ESI mode. It was observed that the signal intensity of positive ion (ESI^+^) was much higher than that of negative ion for both analyte and IS. Parameters, such as capillary and cone voltage, desolvation temperature, ESI source temperature and flow rate of desolvation gas and cone gas were optimized to obtain the optimum intensity of protonated molecular ions (M+ H)^+^ for linifanib and IS. Linifanib produced the maximum intensity of daughter ion signals when cone voltage and collision energy was set at 46 V and 30 eV respectively, whereas for IS the optimized cone voltage and collision energy was 36 V and 22 eV respectively. The MS spectra (parent ions) and MS/MS spectra (product ions) of linifanib and IS are shown in Figure 2.

#### Optimization of chromatographic conditions

Several other RTKs (gefitanib, crazotinib and sunitinib) were tested initially for the selection of appropriate IS. Finally sunitinib was chosen due to its similarity in chemical structure and polarity with linifanib and also high recovery. Binary mixture of volatile buffers (e.g. ammonium acetate and formate) and polar organic solvent like acetonitrile and methanol comprises the majority of mobile phase utilized for liquid chromatography tandem mass spectroscopy and are striking for use due to their miscibility, low viscosity, ability to produce good chromatographic peak shape and compatibility with MS/MS detection. So the feasibility of different compositions of acetonitrile or methanol with ammonium acetate were tried for separation of the analyte and IS with altered flow-rates (in the range of 0.2–0.4 mL/min) on BEH™ C18 column (50 × 2.1 mm, i.d. 1.7 μm).The best peak resolution along with high sensitivity was achieved with an isocratic elution by a mobile phase comprising of acetonitrile: 10 mM ammonium acetate (60:40) at a flow-rate of 0.3 mL/min.

#### Optimization of sample processing

Clean samples are essential to minimize ion suppression and matrix effect in bioanalytical method. For its simplicity, protein precipitation was investigated as the first option for sample preparation. Phospholipids present in plasma are considered as one of the most significant matrixes interferences encounter in protein preparation methodologies [19]. Due to ease and compatibility with mobile phase, protein precipitation using acetonitrile and methanol (with or without acetic acid/formic acid) were tried. Acidification of plasma by formic acid followed by protein precipitation by acetonitrile produced maximum recovery and therefore used for sample preparation.

### Method validation

#### Selectivity

**Figure 3:**
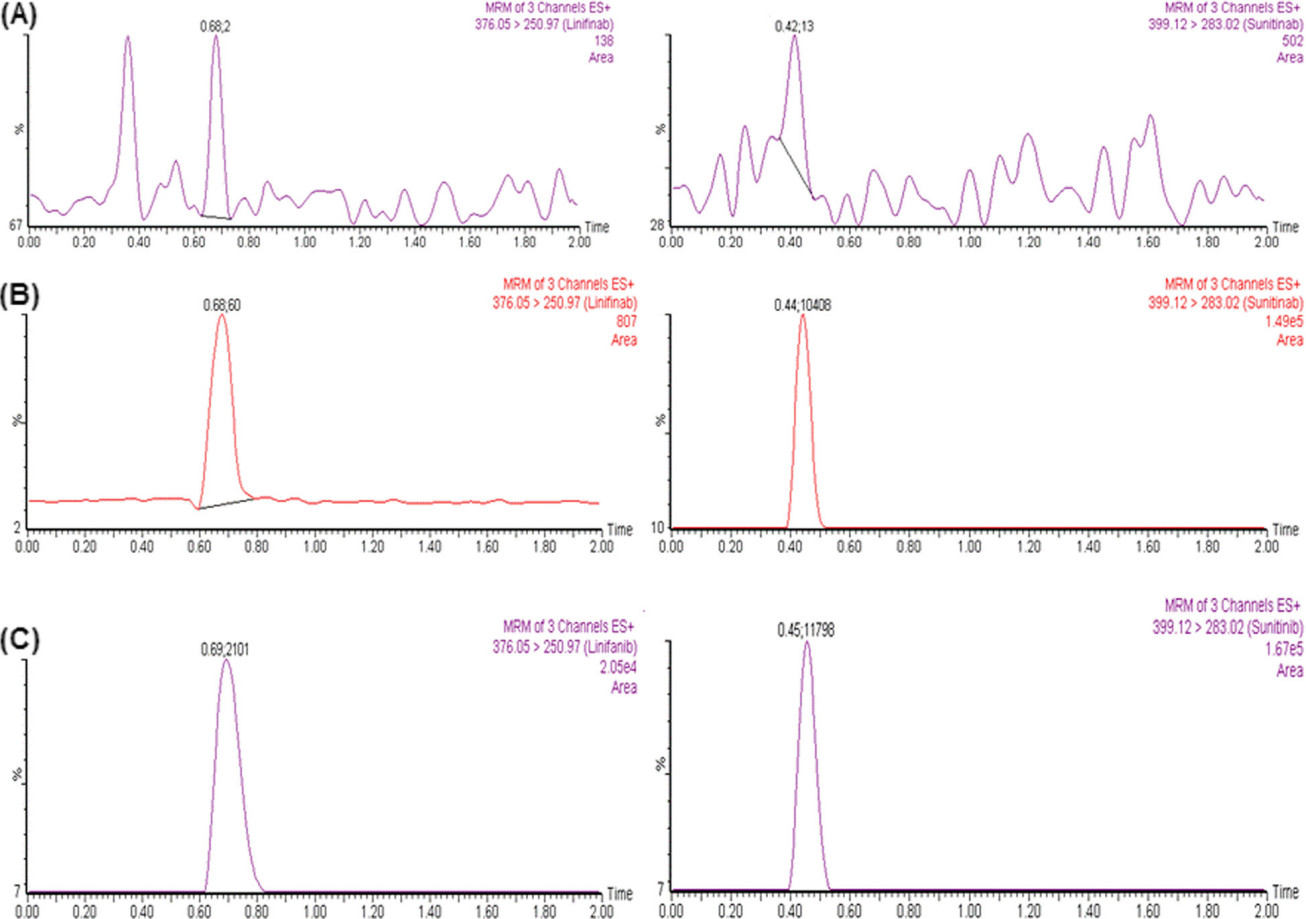
Representative MRM chromatograms of linifanib and IS in (A) blank plasma; (B) plasma spiked at LLOQ level and (C) 1 h after oral administration of linifanib 5 mg/kg in rat.

No peaks (≥20% in comparison to the spiked LLOQ and ≥5% in comparison to IS) were detected in blank plasma (obtained from different rats) at the corresponding retention time of linifanib and IS. This indicates that the method looks to be selective enough for quantification of linifanib. Representative MRM chromatograms of linifanib and IS for blank plasma (showing no significant interference at the retention time of the analyte and IS), plasma spiked at LLOQ level and at 1 h after administration of linifanib are shown in Figure 3.

#### Linearity and lower limit of quantification

The linearity was calculated by weighted least squares regression analysis (1/X^2^) of analyte/IS peak area ratios versus analyte concentration. The method was found to be linear in the range of 0.40-500 ng/mL for linifanib in rat plasma. The correlation coefficients (r^2^) were found to be consistently ≥0.996 during the course of validation. The lowest point on the standard curve was accepted as LLOQ (0.40 ng/mL) for this assay.

#### Precision and accuracy

Accuracy and precision were calculated based on three batches of the four QC samples. The intra- and inter-day precision values were ≤ 10.6 and ≤ 10.4 respectively. Likewise the intra- and inter-day accuracies were in the range of 90.9–108.9 and 92.8 – 106.8%, respectively (Table 1). The precision values should not exceed ±15% and accuracy was required to be within ±15% (20% for LOQ QC).

**Table 1:** Intraday and interday precision and accuracy of linifanib in rat plasma

**Nominal concentration (ng/mL)**	**Run**		**Rat plasma**	
		**Mean ± SD**	**Precision (% CV)**	**Accuracy (%)**
***Intraday variation (six replicate at each concentration)***				
0.45	1	0.48 ± 0.05	10.4	107.0
	2	0.47 ± 0.05	10.6	104.4
	3	0.49 ± 0.05	10.2	108.9
1.28	1	1.35 ± 0.14	10.4	105.3
	2	1.38 ± 0.14	10.1	105.0
	3	1.35 ± 0.08	5.9	105.6
42.5	1	44.5 ± 3.28	7.4	104.6
	2	39.7 ± 3.58	9.0	93.5
	3	40.2 ± 1.56	3.9	94.6
425	1	397 ± 36.4	9.2	93.5
	2	387 ± 12.2	3.2	90.9
	3	398 ± 13.5	3.4	93.9
***Interday variation (18 replicates at each concentration)***				
0.45		0.48 ± 0.05	10.4	106.8
1.28		1.36 ± 0.09	6.6	106.3
42.5		41.48 ± 3.53	8.5	97.6
425		394 ± 22.79	5.8	92.8

#### Recovery and matrix effects

The extraction efficiency of the method was determined by the percentage recoveries of linifanib obtained from plasma at three different QC concentration levels (1.28, 42.5 and 425 ng/mL) and IS (100 ng/mL). The mean percentage recovery was 71.8 ± 9.2% for linifanib and 75.1% for IS (Table 2). These results indicate that the recovery of linifanib using protein precipitation method by acetonitrile was satisfactory, consistent and concentration independent. The matrix effects were examined to assess the possibility of the ionization suppression or enhancement. The data for matrix effects were in the range of ±15%, indicating no significant matrix effects.

**Table 2:** Recovery data of linifanib (three QC samples) and IS in rat plasma (Mean ±SD)

	**Nominal concentration (ng/mL)**	**Percentage recovery**
**Linifanib (Analyte)**	1.28	82.5
	42.5	67.3
	425	65.7
		71.8 ± 9.2
**Sunitinib (IS)**	100.0	75.1

#### Stability and dilution integrity

The stability results of freeze–thaw, post preparative, short-term and long-term are summarized in Table 3. Results indicate that linifanib spiked into rat plasma was stable during three freeze-thaw cycle, at least 6.0 h at room temperature, 48 h in autosampler and up to 30 days at around −80°C. The stock solutions and working standard of linifanib and IS were also stable for 15 days at refrigerator temperature (below 10°C) and for 12 h at room temperature. In dilution integrity study, the percentage accuracy of two and four times diluted samples were 98.9 and 100.7% of the nominal concentration for linifanib. These results concluded that the dilution of the concentrated plasma sample up to four times maintains legibility and integrity of linifanib concentration.

**Table 3:** Stability and dilution integrity data of linifanib in rat plasma

**Stability**	**Nominal concentration (ng/mL) (n = 6)**	**Mean ± SD**	**Precision (% CV)**	**Accuracy (%)**
**Bench top (6 hrs)**	1.28	1.35 ± 0.06	4.4	105.0
	425	393 ± 34.8	8.9	92.7
**Freeze thaw (3 cycle)**	1.28	1.30 ± 0.10	7.7	101.1
	425	385 ± 14.3	3.7	90.7
**In injector (48 hrs)**	1.28	1.42 ± 0.09	6.3	110.6
	425	455 ± 19.8	4.4	107.2
**30 days at −80°C**	1.28	1.26 ± 0.10	7.9	98.3
	425	384 ± 20.2	5.3	90.5
				
**Dilution integrity**	225	226 ± 16.4	7.3	100.7
	450	444 ± 20.6	4.6	98.9

#### Pharmacokinetic study in rats

**Figure 4:**
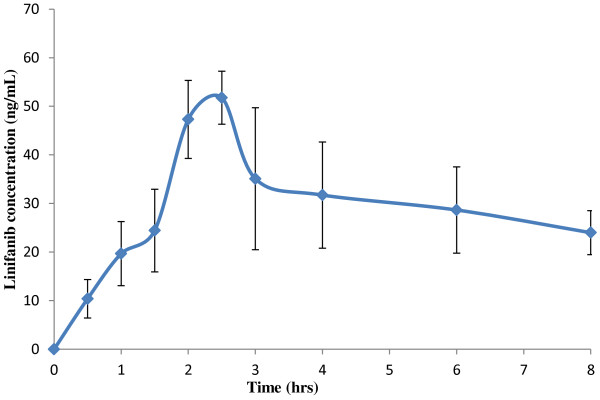
Mean ± SD plasma concentration- time curves of linifanib after a single oral dose of 5 mg/kg in rats.

The developed method was applied to analyse the linifanib concentration in the rats plasma After oral administration of linifanib (5 mg/kg), mean peak plasma concentration of 55.09 ng/mL were achieved after 2.5 hrs. The mean AUC_0-8h_ was 232.05 ng.hr/mL and average t_½_ and K_el_ of 6.67 h and 0.10 hr^-1^ respectively. The mean plasma concentration versus time profile of linifanib in rats is shown in Figure 4.

## Conclusion

A selective and sensitive UHPLC-MS/MS method was developed and validated for the determination of linifanib (ABT-869) in rat plasma for the first time. Compared to previous reported method in human plasma, this method is more sensitive, rapid and simple in terms of chromatographic separation. The developed method was applied to determine the plasma concentrations of linifanib in rats in a pilot pharmacokinetic study. The simplicity and broad calibration range of this assay can be applicable to further characterize linifanib in drug development.

## Abbreviations

UHPLC-MS/MS: Ultra high performance liquid chromatography tandem mass spectroscopy; TKI: Tyrosine kinase inhibitor; VEGFR: Vascular endothelial growth factor recepror; PDGFR: Platelet derived growth factor receptor; LLOQ: Lower limit of quantification; LLOQ QC: Lower limit of quantification for quality control; LQC: Low quality control; MQC: Middle quality control; HQC: High quality control; ESI: Electrospray ionization; MRM: Multiple reaction monitoring; HPMC: Hydroxyl propyl methyl cellulose; Cmax: Maximum plasma concentration; Tmax: Time to C_max_; AUC: Area under curve; t½: Half-life; Kel: Elimination rate constant.

## Competing interests

The authors declare that they have no conflict of interests.

## Authors’ contribution

MI conducted the development and validation of the method, manuscript writing; EE: Performed the pharmacokinetic study and rat samples analysis. NYK: Designed the study and arranged the reference standard; TAW: Assisted in method validation and manuscript writing. All authors have read and approved the final version of the manuscript.
